# CMS121: a novel approach to mitigate aging-related obesity and metabolic dysfunction

**DOI:** 10.18632/aging.205673

**Published:** 2024-03-20

**Authors:** Alcir L. Dafre, Saadia Zahid, Jessica Jorge Probst, Antonio Currais, Jingting Yu, David Schubert, Pamela Maher

**Affiliations:** 1 Cellular Neurobiology Laboratory, Salk Institute for Biological Studies, La Jolla, CA 92037, USA; 2 Neurobiology Research Laboratory, Atta ur Rahman School of Applied Biosciences, National University of Sciences and Technology (NUST), Islamabad, Pakistan; 3 Department of Biochemistry, Federal University of Santa Catarina, Florianópolis, SC 88040-900, Brazil; 4 The Razavi Newman Integrative Genomics and Bioinformatics Core, Salk Institute for Biological Studies, La Jolla, CA 92037, USA

**Keywords:** obesity, diabetes, geroneuroprotection, metabolic disorders, ketogenic diet

## Abstract

Background: Modulated by differences in genetic and environmental factors, laboratory mice often show progressive weight gain, eventually leading to obesity and metabolic dyshomeostasis. Since the geroneuroprotector CMS121 has a positive effect on energy metabolism in a mouse model of type 2 diabetes, we investigated the potential of CMS121 to counteract the metabolic changes observed during the ageing process of wild type mice.

Methods: Control or CMS121-containing diets were supplied *ad libitum* for 6 months, and mice were sacrificed at the age of 7 months. Blood, adipose tissue, and liver were analyzed for glucose, lipids, and protein markers of energy metabolism.

Results: The CMS121 diet induced a 40% decrease in body weight gain and improved both glucose and lipid indexes. Lower levels of hepatic caspase 1, caspase 3, and NOX4 were observed with CMS121 indicating a lower liver inflammatory status. Adipose tissue from CMS121-treated mice showed increased levels of the transcription factors Nrf1 and TFAM, as well as markers of mitochondrial electron transport complexes, levels of GLUT4 and a higher resting metabolic rate. Metabolomic analysis revealed elevated plasma concentrations of short chain acylcarnitines and butyrate metabolites in mice treated with CMS121.

Conclusions: The diminished *de novo* lipogenesis, which is associated with increased acetyl-CoA, acylcarnitine, and butyrate metabolite levels, could contribute to safeguarding not only the peripheral system but also the aging brain. By mimicking the effects of ketogenic diets, CMS121 holds promise for metabolic diseases such as obesity and diabetes, since these diets are hard to follow over the long term.

## INTRODUCTION

The overweight population is increasing around the world, and it is estimated that over a billion people will be obese by 2030 [1]. Obesity is recognized as a disease with severe health impairments, eventually leading to metabolic syndrome, which is characterized by increased values for blood pressure, waist circumference, fasting glucose and high-density lipoprotein levels [2]. The expenses associated with obesity are on the rise. The global cost to health services is estimated to be a staggering US$ 990 billion annually, constituting more than 13% of the entire healthcare expenditure worldwide [1].

In the process of aging, alterations in fat distribution can be accelerated by obesity, with a shift from subcutaneous to visceral fat depots and decreased activity of both brown and white adipose tissue [3]. Age-related alterations in body composition often leads to the simultaneous development of two interconnected conditions: sarcopenia, characterized by a decline in muscle mass, and an increase in fat mass, giving rise to a condition known as osteosarcopenic obesity [4]. This interplay between sarcopenia and obesity has profound consequences for the health of aging individuals, as it has been linked to an elevated risk of immobility, falls, fractures, and other functional impairments, all of which can contribute to higher mortality rates in older adults [5, 6]. Furthermore, these health challenges are compounded by additional factors, such as mood-related phenotypes, like depression [7], and age-associated cardiovascular issues, including hypertension, diabetes, and heart disease [8, 9]. Moreover, a recent study found that in cognitively normal adults at midlife, a higher visceral fat to abdominal fat ratio was associated with an increase in amyloid pathology, especially in the right precuneus cortex, and lower cortical thickness in Alzheimer’s disease-signature areas as well as insulin resistance [10]. These multifaceted factors collectively contribute to the frailty phenotype, which is notably prevalent in Alzheimer’s disease (AD) patients, affecting approximately 30% of this population.

The significance of addressing these complex health concerns is underscored by the global obesity epidemic, primarily driven by diets rich in high levels of fat and sugar coupled with a sedentary lifestyle [11]. In individuals grappling with overweight and obesity, a low-carbohydrate diet, commonly referred to as the ketogenic diet, has shown efficacy in promoting weight loss and reducing triglyceride (TG) levels and diastolic blood pressure. However, it is essential to recognize that implementing dietary changes is ultimately an individual responsibility. Despite the potential benefits of diets like the ketogenic diet, adherence to such regimens frequently presents a significant challenge [12, 13]. As such, addressing these interconnected health issues requires a comprehensive approach that considers not only dietary modifications but also the broader context of aging, body composition, and associated risk factors.

CMS121 was generated by chemical optimization of the flavonol fisetin. It received US FDA Investigational New Drug (IND) approval for testing in humans and we are finalizing the results of a phase 1 clinical trial that looked at its safety and tolerability in healthy young humans (NCT05318040). CMS121 was selected based on its efficacy in protecting against the oxytosis/ferroptosis regulated cell death pathway in HT22 nerve cells [14]. Importantly, when administered to transgenic AD and SAMP8 mice it reduced cognitive decline, and metabolic and transcriptional markers of ageing in the brain [14–16]. By integrating various molecular parameters such as gene expression, metabolites, and proteins, we pinpointed mitochondrial acetyl-CoA as a key metabolite that underlies the neuroprotective effects of CMS121. CMS121 regulates acetyl-CoA metabolism in the brain in a mouse model of accelerated ageing and sporadic AD, preserving mitochondrial homeostasis and promoting histone acetylation at key sites [15]. Notably, these effects on acetyl-CoA metabolism result from the inhibition of fatty acid synthase (FASN) [14], and the inhibition of acetyl-CoA carboxylase 1 (ACC1). Through the activation of 5′ AMP-activated protein kinase (AMPK), ACC1 is inhibited by phosphorylation, limiting the conversion of acetyl-CoA into malonyl-CoA, a limiting step in fatty acid synthesis [15]. Interestingly, this inhibition of fatty acid synthesis was associated with a decrease in free polyunsaturated fatty acids (PUFA) in primary neurons and in the brain [15]. Direct inhibition or knockdown of ACC1 also led to increased acetyl-CoA levels and was itself very neuroprotective against oxytosis/ferroptosis [15].

In addition to its benefits in the ageing brain, CMS121 has a potential to enhance metabolic health in db/db diabetic obese mice. Notable improvements in multiple metabolic parameters, including enhanced glucose metabolism, improved lipid profiles, reduced liver inflammation, and renal protection were observed when CMS121 was administered in the diet [17]. The results of this study suggested that CMS121 might also have benefits in wild type (WT) mice. In the current study, C57BL/6 mice were subjected to a 6-month dietary regimen incorporating CMS121 to comprehensively assess its impact on metabolic activity. We evaluated various facets of metabolic health, encompassing body mass indexes, plasma glucose and lipid levels, as well as metabolomic profiles in WT mice. Additionally, we explored some molecular aspects of its effects by examining metabolic enzyme markers in both adipose tissue and liver. This comprehensive analysis aimed to further understand how CMS121 influences the metabolic landscape, paving the way for potential therapeutic applications beyond its established geroneuroprotective benefits.

## RESULTS

In the course of the study that examined the effects of the CMS121 diet on leptin insensitive db/db mice [17], we noticed that CMS121 presented a clear effect in WT mice regarding glucose and lipid status as well. Therefore, we performed additional analyses to characterize the metabolic changes induced by CMS121 in WT C57/Bl6 mice.

**Figure 1:**
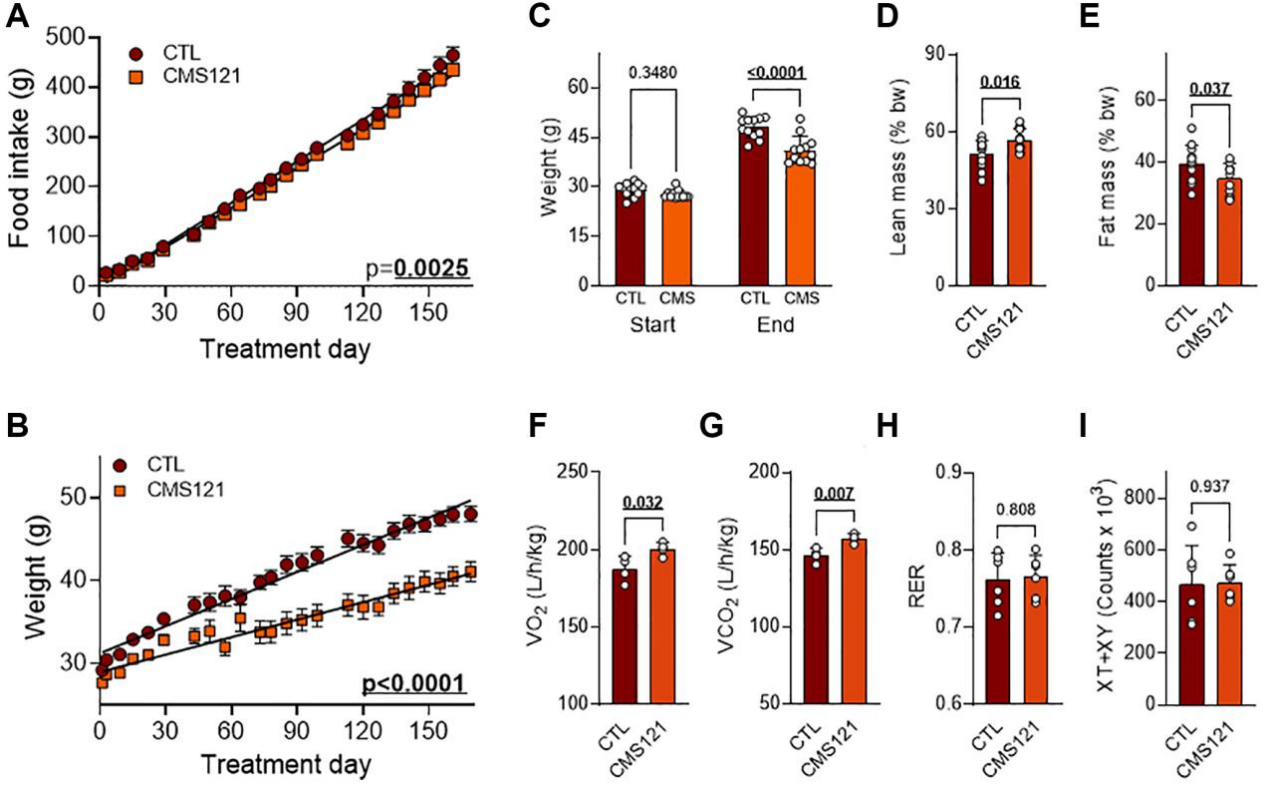
**Nutritional, body mass, locomotor activity, and metabolic activity of mice fed a control diet or a diet containing the geroneuroprotective drug candidate CMS121 for 6 months.** Beginning at the 5th week after birth, mice were either kept on the control diet (CTL) or put on a diet containing CMS121 ad libitum. Cumulative food intake (**A**) and body weight (**B**). Food intake per animal was based on the average food consumption in cages holding 3 mice allocated among a total of 4 cages, and the body weight of 12 mice was followed. Differences between linear regression slopes of CTL and CMS121-treated mice were analyzed and the *p*-values are shown. Initial and final body weights are presented (**C**). During the 13th week of treatment body mass (*n* = 12) indexes were obtained: lean mass (**D**), and fat mass (**E**). Metabolic activity (**F**–**H**) was evaluated at the 15th week of treatment for oxygen consumption (VO_2_) (**F**); carbon dioxide production (VCO_2_) (**G**); respiratory exchange ratio (RER) (**H**), and ambulatory activity (**I**); Data are presented as mean ± SD (*n* = 4). Bold underlined *p*-values indicate statistical differences.

To uncover any possible differences in food intake, linear regressions were compared and found to be different (Figure 1A), indicating a slightly lower food intake in the CMS121-treated mice. Linear regression comparison also indicated that control mice presented a consistently higher weight gain (Figure 1B). Control mice started the experiment weighing 29.2 ± 2.0 g (average ± SEM) at the age of 5 weeks and reached 48.0 ± 3.2 g by the 6th experimental month, with an average weight gain of 18.8 ± 2.6 g (Figure 1C). The starting weight increased from 27.6 ± 1.3 g to 41.1 ± 4.2 g in mice receiving the CMS121 diet (Figure 1C). The lower weight gain of 13.4 ± 2.9 g was statistically significant as compared to the control mice (CTL). In addition, the mice receiving the CMS121 diet presented significantly greater lean mass (Figure 1D), and less fat mass (Figure 1E). The metabolic activity of the mice was also increased by the CMS121 diet with higher oxygen consumption (Figure 1F), and carbon dioxide production (Figure 1G), without an appreciable effect on the RER (Figure 1H) and without affecting the overall locomotor activity (Figure 1I).

**Figure 2:**
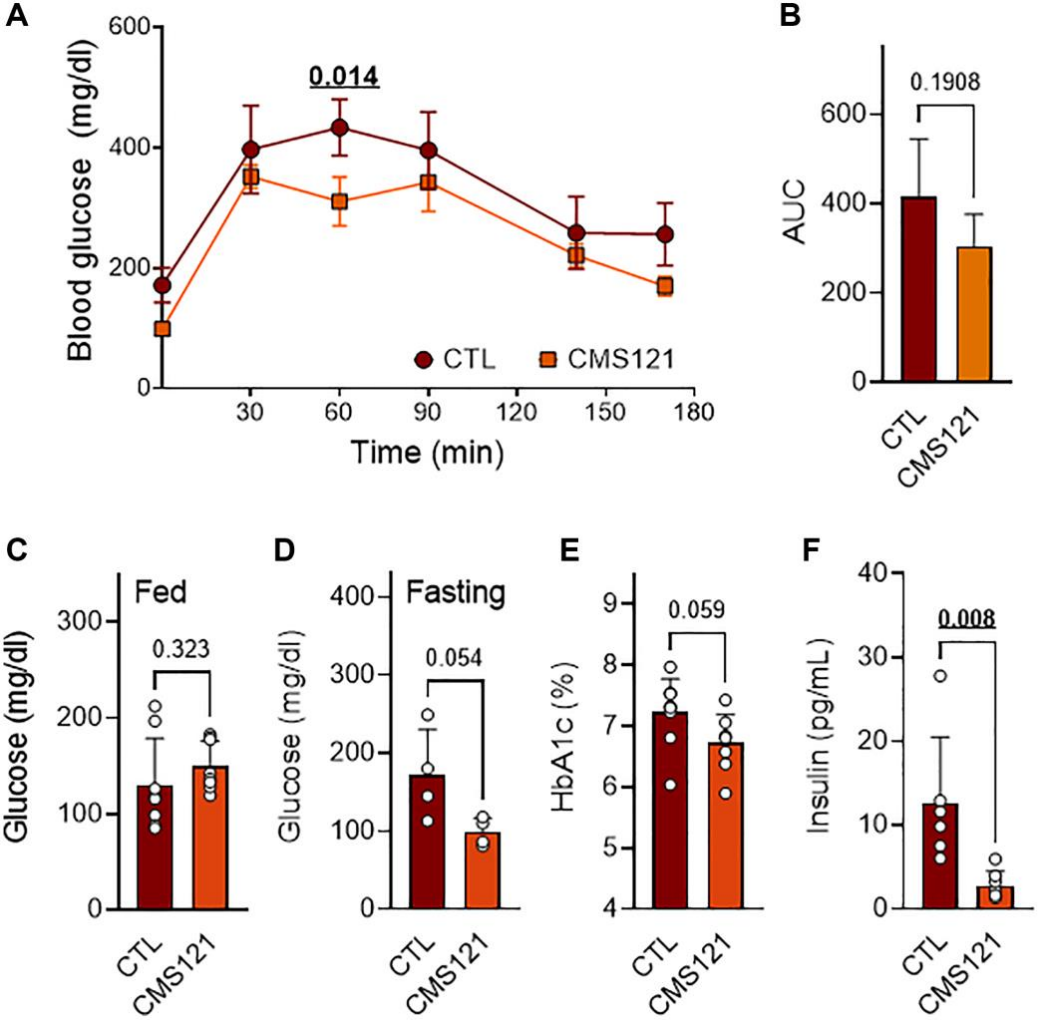
**Glucose status in mice treated with CMS121 for 6 months.** The glucose tolerance test (GTT, *n* = 4–5) was performed at the 6th month of treatment (**A**). The area under the curve (AUC) is presented in (**B**). Glucose was evaluated by caudal vein puncture after the 5th month of treatment in fed mice (**C**) (*n* = 8). At the end of the study, blood was collected for the measurement of fasting glucose (**D**) (*n* = 4), glycated hemoglobin (**E**) (HbA1c; *n* = 8–9), and insulin levels (**F**) (*n* = 6–7). Data are presented as mean ± SD, except for GTT (mean ± SEM). Bold underlined *p*-values indicate statistical differences.

Glucose presented a tendency to lower levels in the glucose tolerance test (GTT, Figure 2A, 2B), and when evaluated in the blood after fasting (Figure 2D), which was not seen when glucose was assessed in fed animals (Figure 2C). In agreement with a lower glycemic status, glycated hemoglobin also presented a tendency to decreased levels (Figure 2E), while insulin levels were markedly decreased (Figure 2F) in the mice fed with the CMS121 diet.

**Figure 3:**
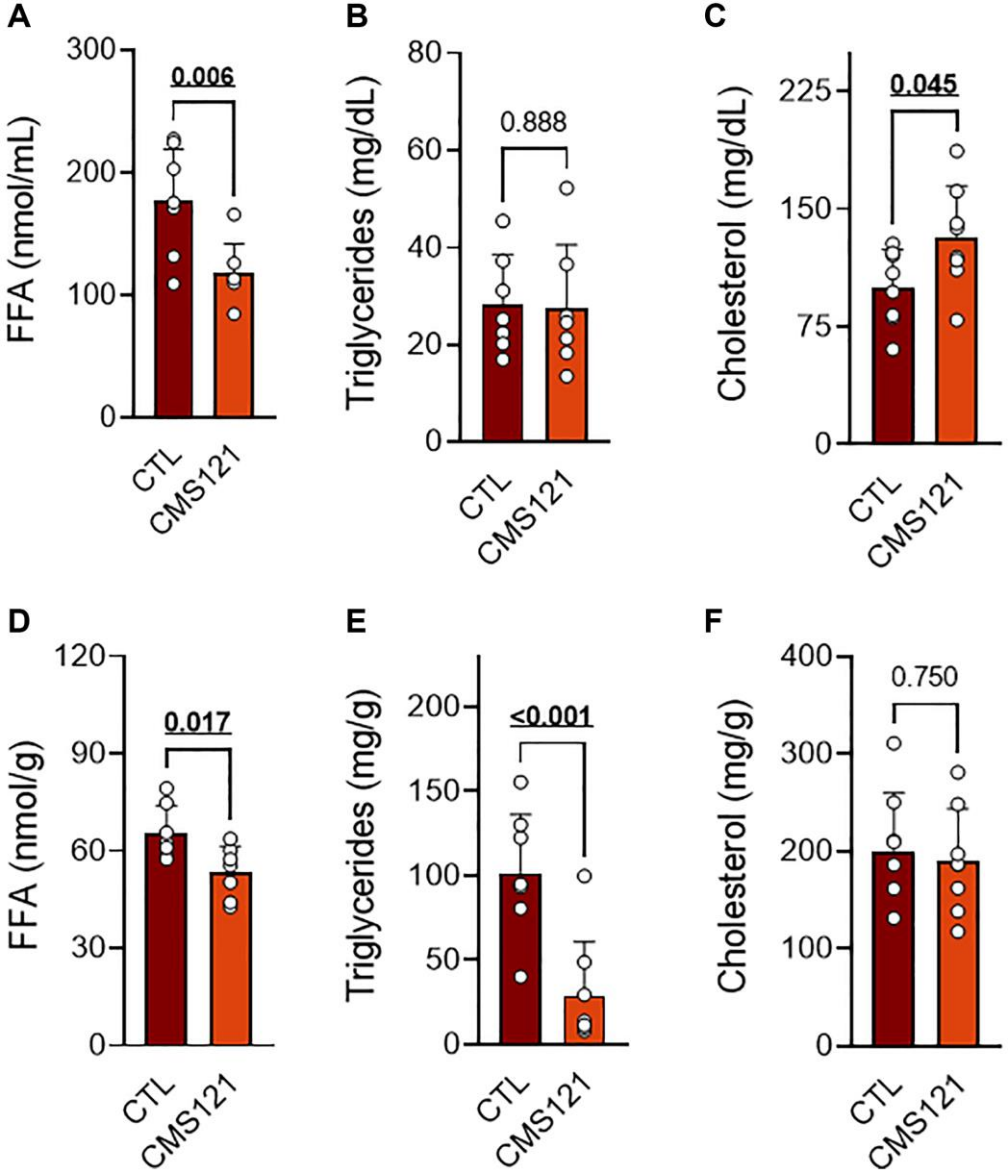
**Blood and liver lipids of mice fed control diet or a diet containing CMS121 for 6 months.** Plasma (**A**–**C**) and liver (**D**–**F**) were evaluated for free fatty acids (FFA (**A**, **D**)), triglycerides (**B**, **E**), and cholesterol (**C**, **F**) at the end of the experiment. Data are presented as mean ± SD (*n* = 7–8). Bold underlined *p*-values indicate statistical differences.

The CMS121 diet also altered the lipid status in blood and liver as compared to the control mice (Figure 3). A significant decrease in free fatty acids (FFA, Figure 3A) was seen in blood, while TG (Figure 3B) remained at control levels, and the cholesterol levels were increased (Figure 3C). A significant decrease in FFA (Figure 3D) and TG (Figure 3E) was also observed in the liver, while cholesterol (Figure 3F) remained unaltered.

**Figure 4:**
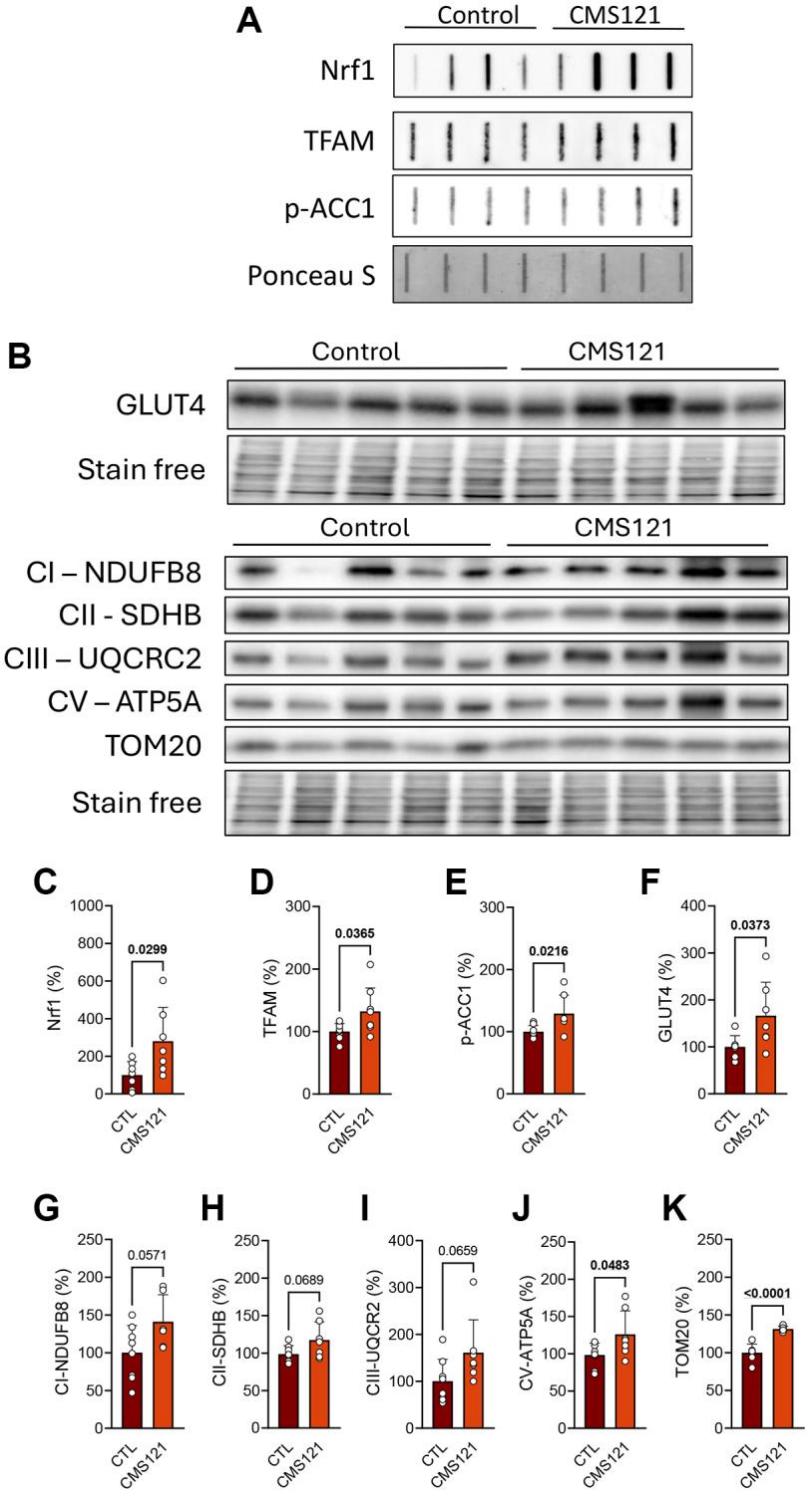
**Adipose tissue metabolic markers were evaluated in mice fed with CMS121 for 6 months.** Representative slot blot (**A**) or Western blot (**B**) images of mitochondrial fractions from adipose tissue are presented. Quantitative results (**C**–**K**) of proteins related to mitochondrial metabolism, such as transcription factors Nrf1 (**C**) and TFAM (**D**), markers of lipid (p-ACC1, (**E**)) and glucose (GLUT4, (**F**)) metabolism, and markers of mitochondrial complex I ((**G**); NDUFB8), complex II ((**H**); SDHB), complex III ((**I**); UQCRC2), and complex V ((**J**); ATP5A) are presented, as well as the levels of the outer membrane translocase TOM20 (**K**). Data are presented as mean + SD (*n* = 6–8). Bold underlined values of *p* indicate statistical differences.

The clear metabolic changes, such as increased lean mass and metabolic activity, allied to lower glucose and lipid status, prompted us to investigate possible mechanisms underlying such alterations. We first investigated the status of mitochondrial proteins, transcription factors associated with mitochondria biogenesis, and markers of lipid and glucose metabolism in the adipose tissue (Figure 4), where representative blot images are presented in panels A and B, and their respective quantifications in C-K. Interestingly, two transcription factors associated with mitochondrial biogenesis were increased by the CMS121 diet, namely nuclear respiratory factor 1 (Nrf1, Figure 4A, 4C) and transcription factor A, mitochondrial (TFAM, Figure 4A, 4D), a response that is in line with an increase in mitochondrial activity, based on the tendency towards increased levels of respiratory complexes I, II, III and V (Figure 4B, 4G–4J). The translocase of outer mitochondrial membrane 20 (TOM20) was also increased by the diet (Figure 4A, 4K). The known inhibitory effect of CMS121 on lipid synthesis [14] was also observed in the adipose tissue, as phosphorylation of ACC1 was increased (Figure 4A, 4E). The levels of the insulin responsive glucose transporter solute carrier family 2 (facilitated glucose transporter), member 4 (GLUT4, Figure 4B, 4F) were also increased by the CM121 diet.

**Figure 5:**
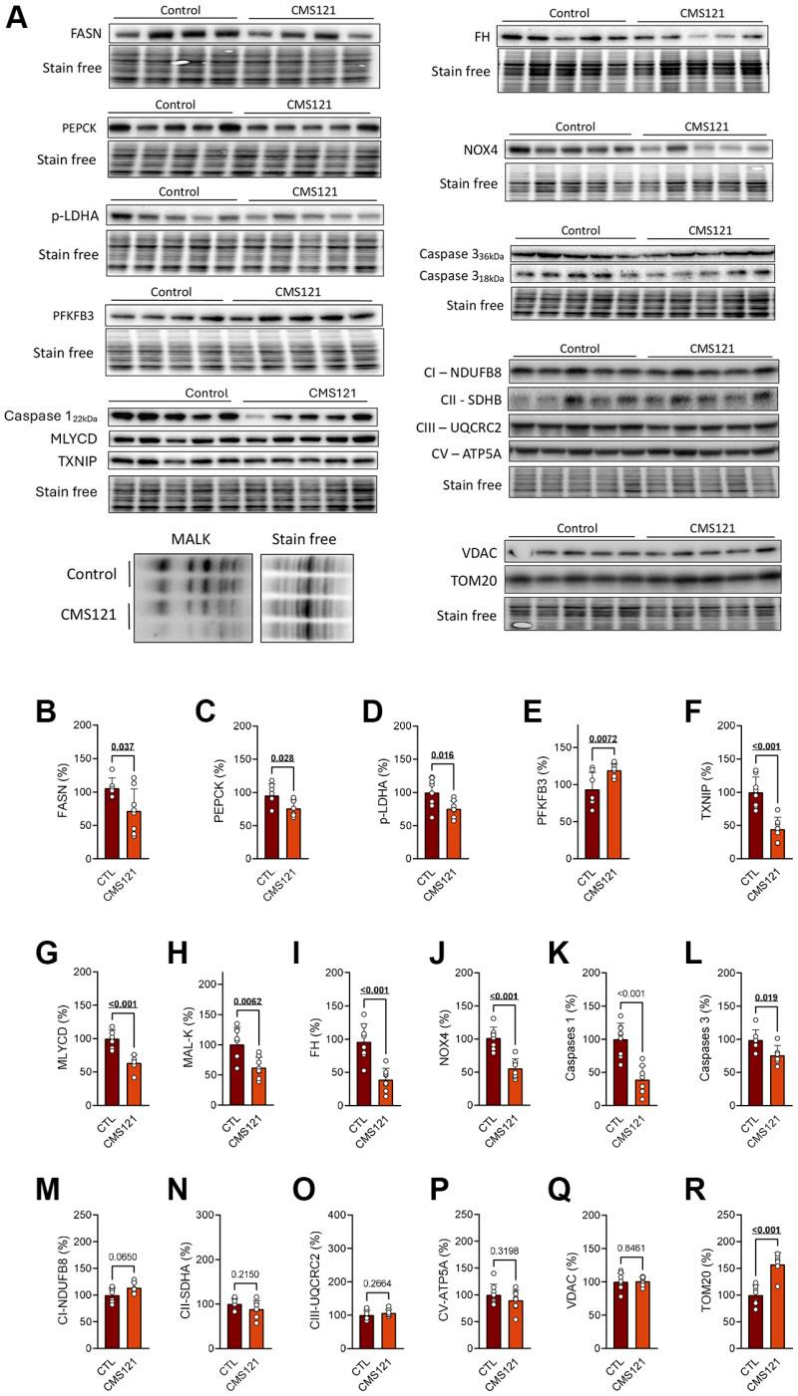
**Liver protein markers were evaluated in mice fed with CMS121 for 6 months.** Representative blot images (**A**), and the corresponding quantification (**B**–**R**) of metabolism markers in the hepatic tissue: FASN (**B**), PEPCK (**C**), p-LDH-A (**D**), PFKFB3 (**E**), TXNIP (**F**), MLYCD (**G**), malonylation of proteins at lysine residues (MAL-K, (**H**)), FH (**I**), Nox4 (**J**), caspase 1 (**K**), caspase 3 (**L**), and markers of mitochondrial complex I ((**M**); NDUFB8), complex II ((**N**); SDHB), complex III ((**O**); UQCRC2), and complex V ((**P**); ATP5A), as well as the voltage dependent anion channel VDAC (**Q**), and the outer membrane translocase TOM20 (**R**). Liver cytosolic fractions were used for blotting, except for the mitochondrial markers (**M**–**R**), FH (**I**), and NOX4 (**J**) that used mitochondrial fractions. Data are presented as mean ± SD (*N* = 6–8). Bold underlined *p*-values indicate statistical differences.

We also examined protein markers of metabolic changes in the liver as shown in Figure 5A, where present representative blot images, and their respective quantification in graphs B-R. The same inhibitory effect on lipid synthesis that was observed in the adipose tissue was also observed in the liver, as indicated by lower levels of FASN (Figure 5B). In addition, the gluconeogenesis marker phosphoenopyruvate carboxylase 1 (PEPCK) was decreased (Figure 5C), indicating decreased gluconeogenesis, while the lower phosphorylation of lactate dehydrogenase A (LDH-A) indicates a decrease in liver lactate production (Figure 5D). An increase in 6-phosphofructo-2-kinase/fructose-2,6-biphosphatase 3 (PFKFB3, Figure 5E) indicates an increase in glycolytic flux, while the decreased thioredoxin interacting protein (TXNIP) levels (Figure 5F) would suggest lower removal of glucose transporters from the plasma membrane, thus favoring glucose uptake. Although the enzyme malonyl-CoA decarboxylase (MLYCD) was decreased by CMS121 (Figure 5G), the concomitant decrease in protein malonylation (Figure 5H) suggests a decrease in malonyl CoA synthesis. The tricarboxylic acid cycle (TCA) enzyme fumarate hydratase (FH) levels were also decreased by CMS121 (Figure 5I).

It was previously noted that CMS121 produced a decreased inflammatory status in the liver of db/db mice [17], and this was also observed in the WT mice receiving CMS121 in the diet, as mitochondrial NADPH oxidase 4 (NOX4) (Figure 5J), caspase 1 (Figure 5K), and caspase 3 (Figure 5L) presented values below those of the control diet group.

None of the mitochondrial respiratory complexes (I, II, III and V) or the voltage-dependent channel were altered in the liver tissue (Figure 5M–5Q), contrasting with the clear increase in the outer mitochondrial translocase TOM20 (Figure 5R), that was seen with the CMS121 diet.

**Figure 6:**
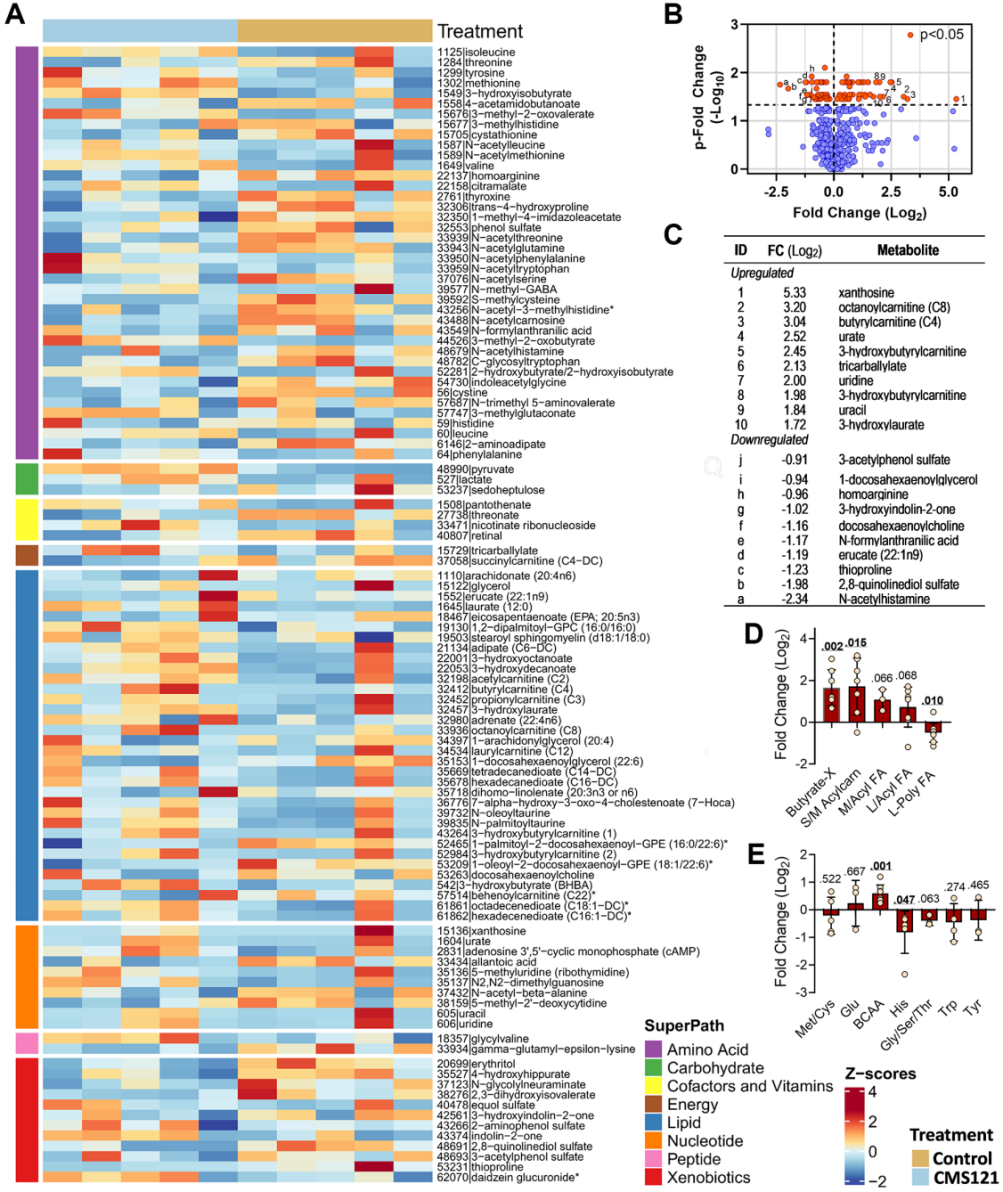
**Effect of the CMS121 diet on plasma metabolites.** Differentially expressed metabolites are presented as (**A**) heat map; (**B**) volcano plot; (**C**) table presenting the top ten upregulated or downregulated metabolites, and metabolites related to lipids (**D**) or amino acids (**E**). Only sub-pathways with 3 or more differentially expressed metabolites are presented in (**D** and **E**), and represent the average ± SD. Abbreviations are: BCAA – branched chain amino acids; Butyrate-X – indicates 4 carbon metabolites related to β-hydroxybutyrate; S/M Acylcarn – small or medium acylcarnitine metabolites; L/Acyl FA – long saturated/monoinsaturated free or acyl fatty acids; L-Poly FA – long polyunsaturated free or acyl-fatty acids. ^*^Indicates compounds that have not been confirmed based on a standard, but mass spectral data were appropriate to reveal their identity.

To gain further insight into the metabolic effects of CMS121, we performed a non-targeted metabolomic study of the mouse plasma. A total of 704 metabolites were identified and 107 (15.2%) of these were altered by the CMS121 diet (Figure 6). Forty-one differentially expressed amino acids represent 38.3% of the total changes. Amino acids, along with 33 lipids representing 30.8% of the total changes, 11 nucleotides representing 10.3% of the total changes and 12 xenobiotics representing 11.2% of the total changes were the most abundant groups presenting differentially expressed metabolites within the super pathways (Figure 6A).

The volcano plot of the results (Figure 6B) shows the metabolites that were significantly influenced by the CMS121 diet (red circles). A similar number of metabolites were found to be increased or decreased by CMS121. When the top 10 increased metabolites (Figure 6C) were analyzed, it was found that acylcarnitine (4) and nucleotide (4) metabolites were the major groups. The top 10 decreased metabolites (Figure 6C) include 4 xenobiotics (3-acetylphenol sulfate, 3-hydroxyindolin-2-one, 2,8-quinolinediol sulfate, thioproline), and 3 lipids (docosahexaenoylcholine, 1-docosahexaenoylglycerol, and erucate).

Although one of the most abundant groups of metabolites influenced by CMS121 are the amino acids, there is no specific pattern of changes when they are analyzed as a group (Figure 6E), except for an increase in the branched chain amino acids (BCAA), and a decrease in the levels of histidine metabolites. The subpathways Met/Cys/SAM/Tau, Glu, Gly/Ser/Thr, Trp and Tyr presented a limited number of metabolites that were altered by the CMS121 diet, precluding further inferences. Interestingly the increase in BCAAs is consistent with an increased mobilization of protein for the production of TCA intermediates and ketone bodies [18], but further experimentation on this topic is required to confirm this idea.

The CMS121 diet also resulted in increased plasma levels of the degradation products of purine (xanthosine) and pyrimidine (uridine and uracil) nucleotides, as well as increased levels of uric acid (Figure 6C). Increased ingestion or catabolism of nucleotides, and/or decreased salvage pathway activity can lead to increased levels of uric acid. Another possible mechanism is increased plasma levels of lactate, which leads to increased renal reabsorption of filtered urate [19]. Nevertheless, purine metabolism is tightly regulated, as is uric acid production and excretion. Elevated concentrations of uric acid are frequently found in subjects consuming a ketogenic diet [20, 21], although the mechanisms underlying this effect were not described. Therefore, any increase in purine catabolism or decreased excretion would promote the accumulation of purine metabolites and uric acid. Without further experimentation, it is not possible to have a clear interpretation of these findings.

When the lipids were grouped, a clear pattern emerged (Figure 6D). Metabolites related to β-hydroxybutyrate (BHB) and acylcarnitines were increased, which is consistent with increased ketone metabolism. Monounsaturated or saturated fatty acids presented a tendency to increase as a group (*p* = ~0.067), but without statistical significance, while the group of PUFA presented a significant decrease.

## DISCUSSION

The CMS121 prototype molecule fisetin protected from high fat diet-induced nephropathy demonstrating clear metabolic effects such as preventing non-alcoholic fatty liver disease, decreasing hepatic fat, increasing muscle mass, and inducing adipose tissue browning [22–24]. CMS121 presented related effects, such as a clear anti-diabetic effect in the db/db obese mouse [17], indicating its potential as a possible treatment for metabolic diseases. Better glycemic and lipid status, decreased liver inflammation, and renal protection were the main effects of CMS121 found in the db/db mice [17]. Here, the potential metabolic effects of CMS121 were explored in WT mice (C57/Bl6) treated for 6 months with CMS121 in the diet.

During normal ageing, locomotor activity declines with progressive body weight gain [25–27], indicating that physical activity is an important health factor [28]. In this regard, laboratory mice have lifelong food availability and generally a sedentary lifestyle. The WT mice in the C57/BL6 background have an average body weight of 33.3 ± 2.8 g at 180 days of age [29]. The same average body weight of C57/BL6 mice at 6 months of age, as reported by the Jackson Laboratory, is frequently observed in the literature [30–33]. However, in our study, the final weight of the WT mice (48.0 ± 3.2 g) indicates that the mice became overweight over the experimental period. The reasons for this weight gain in the WT C57/BL6 mice above what is reported by the Jackson Laboratory, are not clear and warrant further experimentation. Nevertheless, it allowed us to investigate the effects of CMS121 on age-dependent weight gain. The final body weight of mice given the CMS121 diet (41.1 ± 4.2 g) approached the literature values but remained elevated above the expected values (~30–35 g), despite a 40% lower weight gain (13.4 ± 2.9 g) as compared to untreated controls (18.8 ± 2.6 g). We hypothesize that the lower weight gain likely contributed to the improved glucose and lipid status of the CMS121-treated mice. Besides being lighter, the mice given the CMS121 diet also had more muscle and less fat, as well as an increased metabolic rate under basal conditions.

A slight decrease in food consumption cannot explain the much lower weight gain in the mice receiving CMS121. Locomotor activity was not altered by the diet, as evaluated at the 15th treatment week in metabolic cages. However, the limited number of animals evaluated and the stressful condition of the metabolic cage precludes a definitive conclusion. In this regard, two weeks after the diet was introduced to the mice, locomotor activity was increased in the open field test (Supplementary Figure 1), keeping open the possibility that CMS121 can induce an increase in locomotor activity, which certainly would be relevant to the observed metabolic improvements. Unfortunately, we do not have a later evaluation of locomotor activity in the open field test. Despite only finding a statistical decrease in glucose levels at 60 min in the GTT, there is a clear tendency to lower glucose levels during fasting. This trend is reinforced by a tendency to lower levels of HbA1c and markedly decreased levels of insulin in the mice receiving CMS121. If the lipid profile is included, with lower FFA and TG in liver and lower FFA in blood, it comprises a picture indicating that CMS121 may bring about several metabolic improvements that were also seen in the db/db mice treated with the same CMS121 diet [17].

The increased GLUT4 levels in the adipose tissue may indicate an increase in insulin sensitivity which is corroborated by the low basal levels of insulin and may contribute towards improved glucose tolerance and lower plasma levels of glucose. Obesity leads to general mitochondrial dysfunction, including decreased mitochondrial mass and downregulated biological function and biogenesis in adipose tissue [34]. The observed increase in transcription factors associated with mitochondrial biogenesis (Nrf1 and TFAM) in adipose tissues, in response to the CMS121 diet, is in line with the observed increase in mitochondrial markers, thus, possibly counteracting the limitation in adipocyte respiration in the heavier WT mice. Increased mitochondrial activity in adipose tissue may also contribute to the observed increase in oxygen consumption, as detected in the metabolic cage analysis, however locomotor activity as a factor cannot be excluded.

Decreased fatty acid synthesis was indicated by increased phosphorylation of ACC1 in adipose tissue, which leads to its inhibition [35] and a decrease in lipogenesis. Plasma metabolomic data showed increased plasma levels of short chain acylcarnitines and several butyrate metabolites, which are typically found following consumption of a ketogenic diet [36]. Interestingly, BHB can be produced by non-canonical sources such as adipocytes [37].

Fatty acid synthesis inhibition was previously associated with a decrease in free PUFA in primary neurons and in the brains of transgenic AD mice [14], decreased FFA in the plasma of db/db obese mice [17], and in the cerebral cortex and plasma of the ageing accelerated SAMP8 mice [15]. Here, these findings are extended to the plasma of CMS121-treated WT mice, as lower FFA levels were seen in the plasma and liver, and lower PUFA in the plasma. PUFA peroxidation is considered an essential component of cell death dependent on oxytosis/ferroptosis [38]. It would be interesting to know how the peripheral PUFA interacts with the brain, and if the lower plasma levels of PUFA represent a possible mechanism of protection against oxytosis/ferroptosis in peripheral tissues, such as the liver and adipose tissue.

Increased lipid mobilization promotes fatty acid oxidation leading to higher acetyl-CoA levels in hepatocytes [39], which is the substrate for the formation of ketone bodies, such as acetoacetate and butyrate metabolites that were found increased in the plasma of mice on the CMS121 diet. A ketogenic diet is known to drastically decrease hepatic TG [40], which was also observed with the CMS121 diet. Reductions in liver fat and *de novo* lipogenesis were also observed in humans on a ketogenic diet [41], in line with the decreased FASN levels found in the liver of CMS121-treated mice. Interestingly, a ketogenic diet can also increase the hydrolysis of hepatic lipids which was associated with lower serum insulin levels, and increased glucose tolerance [42], in agreement with the lower plasma insulin levels and improvements in the glucose status found in mice on the CMS121 diet.

Ketogenic metabolism is well-known for promoting health, particularly in obese and elderly individuals [43, 44]. Several studies also showed benefits for middle aged rodents suggesting that consumption of a ketogenic diet in middle age can reduce the ageing and neurodegenerative effects of an unhealthy diet. Moreover, there are a growing number of preclinical and clinical studies showing that a ketogenic diet is beneficial in AD by numerous mechanisms such as the anti-aging effect of BHB, improved mitochondrial function, changes in the gut microbiota, reduced neuroinflammation, and decreased oxidative stress [44–47]. CMS121 seems to limit fatty acid synthesis by both an inhibitory effect on FASN and by activating AMPK, thus increasing ACC1 inhibition by phosphorylation [14]. Increased ACC1 phosphorylation was observed in adipose tissue and decreased levels of FASN were observed in the liver, both of which would lead to increased levels of acetyl-CoA [39] indicating CMS121 induces the same inhibitory effects on lipid synthesis in hepatic and adipose tissue as found in the brain [14]. Interestingly, CMS121 has been shown to be neuroprotective in ageing and Alzheimer’s disease models, possibly by increasing in acetyl-CoA levels in the brain [15].

The investigation into metabolic reprogramming by CMS121 in hepatic tissue also revealed that gluconeogenesis is probably diminished, as evidenced by lower levels of PEPCK, while the increased levels of PFKFB3 suggest increased glycolysis. Through the production of fructose 2,6-bisphosphate, PFKFB3 is a known activator of PFK1 triggering aerobic glucose oxidation [48]. Increased glycolysis could also be promoted by the decreased expression of TXNIP in the livers of the CMS121-treated mice. TXNIP is a known arrestin that promotes the recycling of glucose receptors, leading to decreased glucose uptake [49]. The lower phosphorylation of LDH-A decreases its activity [50] and would also favor glucose-mediated respiration in the livers of CMS121-treated mice. Nevertheless, the CMS121 diet did not alter the expression of ETC markers which normally would be expected in a scenario of decreased lactate production and increased glycolysis, showing that the effect of CMS121 on liver metabolism is complex and requires further investigation.

The observed increases in the plasma levels of pyruvate and lactate after consumption of the CMS121 diet are also compatible with the idea of increased lactate production and release to the circulation [51]. Given that hepatic LDH-A phosphorylation was decreased, suggesting an inhibition [52], it is possible that the origin of the plasma lactate is extra-hepatic. In the traditional view, increased lactate levels are taken up by the liver and passed to the Cori cycle to increase glucose production and its release to the blood, which spares glucose to extrahepatic tissues, and thereby limits protein degradation [53]. However, pyruvate and lactate can directly enter the TCA cycle and are readily oxidized by skeletal muscle during rest and contraction [54], reinforcing the notion that circulating lactate can be a primary source of carbon for the TCA cycle and thus a valuable energy source [50, 55]. Furthermore, the overall picture indicates that mice receiving CMS121 are in a metabolic state resembling that produced by a ketogenic diet which is also consistent with preferential fatty acid oxidation usually found during anabolic metabolism [53]. The increased lean mass and decreased fat mass induced by the CMS121 diet is an interesting outcome that could be explored further in the context of the age-related decline of skeletal muscle in osteosarcopenic obesity patients [4].

A deficiency in FH was reported to strongly protect against obesity, insulin resistance, and fatty liver [56]. Conversely, increased FH activity impairs the antioxidant response in obesity [57]. Itaconate is known to modulate the immune and antioxidant responses [58], and miR-144, by preventing itaconate synthesis, increases FH activity, potentially contributing to non-alcoholic fatty liver disease [57, 59, 60]. Elevated levels of fumarate due to lower levels or activity of FH drives the reversal of mitochondrial complex II, promoting the conversion of fumarate to succinate [58, 59], which was also observed during renal β-oxidation of long- and medium-chain fatty acids [61]. Succinate accumulation leads to increased itaconate levels, which can promote fatty acid oxidation in hepatocytes [59]. The CMS121 diet induced a decrease in the hepatic levels of mitochondrial FH, indicating fumarate may be elevated, and potentially contribute to a shift towards ketogenic metabolism that is basically fueled by fatty acids in the liver [43]. Therefore, a decrease in FH levels and an increase in β-oxidation can eventually lead to increased itaconate synthesis, a plausible mechanism that could contribute to the promotion of metabolic remodeling by CMS121. However, additional experiments are needed to clarify this possibility.

Despite limited succinyl-CoA-3-oxoacid CoA transferase activity in hepatocytes and the observation that certain ketone supplements can limit the availability of mevalonate for cholesterol synthesis [62], increased cholesterol is frequently found in studies using a ketogenic diet [63]. The readily available acetyl-CoA resulting from a ketogenic diet can be diverted to the mevalonate pathway, leading to cholesterol synthesis [64]. The elevated levels of plasma cholesterol in mice treated with CMS121 suggests an increase in synthesis, which is consistent with the increased plasma levels of 7-HOCA, a cholesterol metabolite [65]. In addition, limitation of hepatic FASN activity impairs the retention of cholesterol in the plasma membrane [66], which may constitute another possible explanation for the elevated cholesterol levels in the plasma of mice receiving the CMS121 diet. Interestingly, the limited cholesterol availability in the plasma membrane of FASN-deficient cells was associated with a lower inflammatory status, since cholesterol is required for propagating inflammatory signals [66]. The lower hepatic levels of FASN may also account for the decreased inflammatory status found in the livers of mice fed a CMS121 diet. The results showed a clear decrease in hepatic caspase 1, caspase 3 and mitochondrial NOX4, all known to promote hepatic inflammation [67–69]. The obese db/db mice fed a CMS121 diet also showed a clear decrease in hepatic inflammatory markers such as NF-κB, IL-18, and caspase 3 [17], reinforcing the liver anti-inflammatory response induced by CMS121 in mice. The precise mechanisms underlying these anti-inflammatory effects of CMS121 in the liver remain to be determined.

## CONCLUSIONS

Given the growing evidence that dyshomeostasis of peripheral metabolism may play a key role in age-related diseases, the effects of CMS121 on liver and adipose tissue described here suggest a new approach to treating age-related neurodegenerative diseases whereby drug candidates are selected which positively modulate not only brain metabolism but peripheral metabolism as well. Furthermore, based on the results reported here, along with the previously reported metabolic improvements in the db/db obese mice, CMS121 applicability could be expanded from a geroneuroprotector drug to a metabolic drug with special potential to be explored in non-alcoholic fatty liver disease. The metabolic reprogramming towards ketogenic metabolism suggests that CMS121 could be a promising treatment for weight loss and other conditions where a ketogenic diet has shown benefits, especially since these types of diets are hard to follow over the long term.

## MATERIALS AND METHODS

### Animals and treatment

At 5 weeks of age, male C57BL/6 male mice were started on a standard rodent diet (LabDiet 5015, TestDiet, Richmond, IN, USA) with or without CMS121 for 6 months. Mice (12 per group) were assigned randomly to the CMS121 diet or the control diet and housed with 3 mice per cage. Body weight and food consumption were evaluated weekly. Based on the cage average food consumption, the CMS121 ingestion was 9.4 mg/kg/day during the first 17 weeks of treatment (200 ppm) and 18.8 mg/kg/day CMS121 for weeks 18–24 (400 ppm). CMS121 at the indicated doses was previously shown to be effective in mouse models of ageing and AD [14–16] and diabetes [17]. Behavioral testing and data collection were performed with the researcher blinded to the treatment. Body mass and metabolic status were evaluated at the 13th and 15th weeks of treatment, respectively. After 6 months of treatment, animals were sacrificed, and blood, liver, and subcutaneous adipose tissue were collected for further analyses.

### Metabolic evaluation

Echo-MRI analysis was performed at the 13th treatment week to evaluate body mass using an EchoMRI 100 apparatus (Echo Medical Systems, Houston, TX, USA). At the 15th week of treatment animals were placed for 5 days in a metabolic cage system apparatus (LabMaster, TSE-Systems Inc., Chesterfield, MO, USA) equipped to detect indirect calorimetry, measure food and water intake, and monitor activity. Metabolic parameters, such as oxygen consumption (VO_2_), carbon dioxide production (VCO_2_), respiratory exchange rate (RER), and energy expenditure were obtained and data analyzed with CalR software [70].

### Glucose and lipid status

The enzymatic Infinity Glucose Hexokinase Liquid Stable Reagent (Thermo Scientific, Middletown, VA, USA) or Accu-Check Aviva test strips (Roche, Indianapolis, IN, USA) were used to assay blood and tail blood glucose, respectively. Hemoglobin A1c (HbA1c) was measured by an enzymatic assay, and insulin by ELISA, both from CrystalChem (Elk Grove Village, IL, USA). Triglycerides (TG), and plasma cholesterol were assayed by commercial kits (Pointe Scientific, Canton, MI, USA). Liver cholesterol was assayed with a fluorometric assay (Cell Biolabs, San Diego, CA, USA). Free fatty acids (FFA) were evaluated by a fluorescent method (BioAssays Systems, Hayward, CA, USA).

Mice were deprived of food overnight to perform the glucose tolerance test (GTT) on the next day. A glucose solution was given by gavage (0.5 g/kg), as previously described [71]. Blood glucose was sampled at 0, 30, 60, 90 and 120 min by tail vein puncture and evaluated by Accu-Check Aviva test strips (Roche, Indianapolis, IN, USA).

At the end of the experiment, mice were anesthetized, and their blood collected by cardiac puncture. Red blood cells and plasma were separated by using Microvette CB 300 K2E blood separation tubes (Sarstead, Nübrecht, Germany). After perfusion with phosphate buffered saline, adipose tissue and liver were removed and stored at −80°C until further use.

### Western blotting and slot blot

Tissue samples were mechanically homogenized with a TissueRuptor (Qiagen, Hilden, Germany) at a 1:5 w/vol in mitochondria buffer (Hepes 5 mM, EGTA 1 mM, mannitol 220 mM, sucrose 70 mM) with added Roche (Indianapolis, IN, USA) protease and phosphatase inhibitors cocktail. After centrifugation (800 g, 10 min, 4°C), the supernatant was centrifuged at 10000 g for 10 min at 4°C, and the supernatant was considered as the cytosolic fraction, while the pellet was the mitochondrial fraction. Protein was assayed by the BCA method (Invitrogen, Waltham, MA, USA).

Samples were prepared in sample buffer (66 mM Tris/HCl pH 6.8, 10% glycerol, 2% SDS, 2% 2-marcaptoethanol, 0.1% bromophenol blue) and analyzed by SDS-PAGE using 4–12% gradient gels (Criterion XT Precast Bis-Tris Gels, Bio-Rad, Hercules, CA, USA). Proteins were transferred to polyvinylidene fluoride membranes and probed with the desired primary antibody. Stain-free gels were used to quantify proteins (Bio-Rad). Horseradish peroxidase-conjugated secondary antibodies were diluted 1/5000 in 2% skim milk in Tris buffered saline/0.1% Tween 20 (TTBS) prior to use. Images were obtained by the Chemidoc MP Imaging System (Bio-Rad) and quantified with the ImageJ software (https://imagej.nih.gov/ij/).

We have previously validated a few antibodies for slot blot analysis and therefore, for these antibodies, this technique was used instead of Western blots. A nitrocellulose membrane was pre-wet in TTBS (150 mM NaCl, 25 mM Tris/HCl pH 7.4, 0.5% Tween 20) and assembled along with the 2 filter papers (#1620161, Bio-Rad, Hercules, CA, USA) in the Bio-Dot SF apparatus. To wash the membrane, 500 μL of TTBS was passed through the membrane by suction and kept under negative pressure for 10 min to completely remove the TTBS. Sample (20 μL) was applied directly to the membrane using a multichannel pipette and kept for 15 min in a fume hood to allow the samples to pass through the membrane. Membranes were washed with deionized water for 5 min until the bromophenol blue was completely removed, and soaked in a fixative solution (50% methanol, 6% acetic acid, and 44% water), and kept under agitation for 5 min on a rocking shaker. After a 5 min wash in TTBS, samples were incubated with the primary antibody. The remaining procedures were identical to those used in Western blotting. Ponceau S staining was used as the loading control.

The following antibodies were used: caspase 1 (#4199), caspase 3 (#9661), TOM20 (#42406), VDAC (#4866), ACC1 phosphorylated at serine 79 (#3661), GLUT4 (#2213), FASN (#3189), LDH-A phosphorylated at tyrosine 10 (#8176), PFKFB3 (#13123), TXNIP (#14715), MAL-K (#14942), FH (#4567) were from Cell Signaling (Danvers, MA, USA); Nrf1 (#175932), TFAM (#131607), mitochondrial electron transport chain (ETC) markers (#110413) including complex I (CI, NDUFB8), CII (SDHB), CIII (UQCRC2), CV (ATP5A) were obtained from Abcam (Waltham, MA, USA); NOX4 (#C313066) was obtained from Life Span Biosciences (Mandideep, India); phosphoenopyruvate carboxylase 1 PEPCK1/PCK1 (#10004943) was obtained from Cayman (Shirley, MA, USA); MLYCD (#pro15265-1-ap) was obtained from Proteintech (Rosemont, IL, USA).

### Metabolomic analyses of plasma samples

Metabolomic analysis was performed at Metabolon with plasma samples from an independent experiment, as previously published [14]. Raw data are available in Supplementary Table 1. At 9 months of age, male controls (C57BL/6J) were fed a diet (Harlan Teklad; Envigo, Indianapolis, IN, USA) with or without 400 ppm CMS121, leading to an average consumption of 34 mg/kg/day CMS121, based on the measurement of the overall food consumption of the mice housed together in each cage. Treatment lasted for 3 months, and mice were assigned randomly to treatment or control groups and data analysis was performed by different blinded researchers. The sample preparation and initial analysis were done by Metabolon, as previously published [14]. Datasets with 3 or more missing values were excluded from the analysis, as well as outliers. The outliers were identified using the interquartile range (IQR) of data, which is the range between the first (Q1) and the third (Q3) quartiles (IQR = Q3 – Q1). The data points which fall below Q1 – 1.5 × IQR or above Q3 + 1.5 × IQR are defined as outliers. After filtering out 10 metabolites, from a total of 714 metabolites, 107 presented differences between control and CMS121-treated mice. Differential test was performed by the Wilcoxon rank-sum test and considered significant when *p* < 0.05.

### Statistical analyses

Weight and food intake data were adjusted to a linear regression and slopes of linear regressions were compared. Pairwise samples were compared by the student’s *t* test and significant differences were set at *p* < 0.05. For the GTT, two-way ANOVA was used followed by the Bonferroni *post hoc* test for multiple comparison analysis. Unless stated, data are presented as average ± SD, and sample number is indicated in the figure legends.

### Availability of data and materials

The datasets generated and/or analyzed in the current study are available from the corresponding authors upon reasonable request.

## Supplementary Materials

Supplementary Figure 1

Supplementary Table 1
