# Supplementary material for: CMS121: a novel approach to mitigate aging-related obesity and metabolic dysfunction
Source: Aging (Albany NY). 2024 Mar 20;16(6):4980–99. doi: 10.18632/aging.205673 (PMC11006478; doi:10.18632/aging.205673)
Supplement: Supplementary Figure 1 [file aging-16-205673-s001.pdf]

SUPPLEMENTARY FIGURE

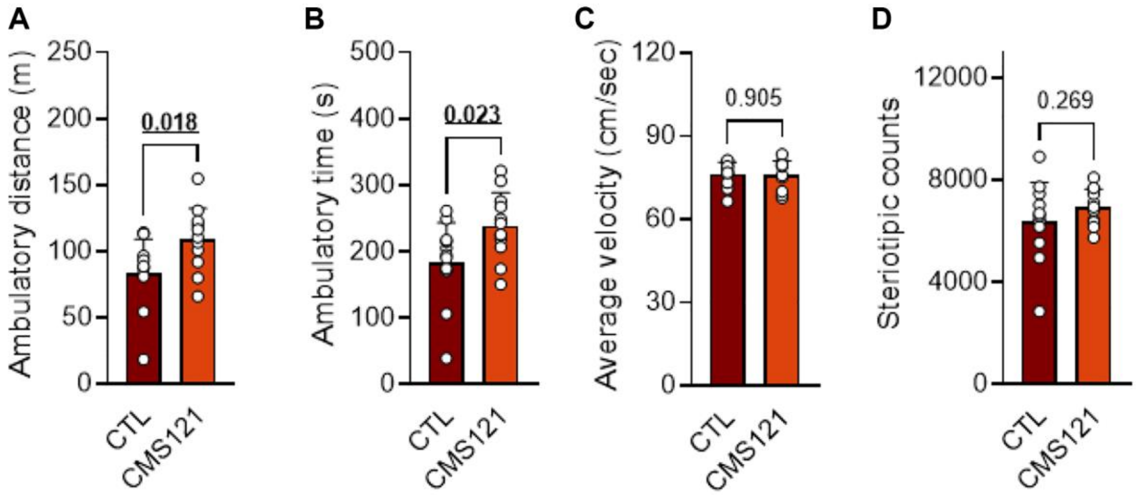

Supplementary Figure 1. Locomotor activity in animals freely exploring an open field two weeks after treatment began. Animals were exposed to the open field for 5 min and their activity recorded and analyzed with the Anymaze software. (**A**) Total distance traveled; (**B**) ambulatory time, (**C**) average velocity, and (**D**) stereotypic movements. Data are presented as mean + SD (n = 12). Bold underlined p-values indicate statistical significance.
